# Supplementary material for: Patterns of Intron Gain and Loss in Fungi
Source: PLoS Biol. 2004 Nov 30;2(12):e422. doi: 10.1371/journal.pbio.0020422 (PMC532390; doi:10.1371/journal.pbio.0020422)
Supplement: Table S1 — Also available at http://genes.mit.edu/NielsenEtAl/. (4.3 MB ZIP). [file pbio.0020422.st001.zip › NielsenEtAl/html/739.html]

AN0451.1.NCU04156.1.MG08624.1.FG07315.1


```
 CLUSTAL W (1.82) Multiple Sequence Alignments - Introns Inserted


Sequence 1: MG08624.1	221 aa
Sequence 2: FG07315.1	235 aa
Sequence 3: AN0451.1	220 aa
Sequence 4: NCU04156.1	285 aa
Alignment Length: 290 aa
Number Identitical Residues: 134 aa
Alignment Score (without introns) 6089


MG08624.1 	--------------------------MSSP---------ISGFLRFVAVLLAVVSPLVYL
NCU04156.1	MPPKKQSSSGGNKPSGSGSSSGRSSSGSSCRCSCRCRCSIGGWLKFFAILFALVAPIAYV
FG07315.1 	-------------------MAKTKSKSSKPSSASKSQGGISKLILVLGLLTALLSSVVYF
AN0451.1  	---------------------------------MRC-------LSIAVAFLALFTGLYRF
          	                                   .       : .   : *:.: :  .

MG08624.1 	AEQRLESFYVFDHKHLHELSTQAIAQHGNNTRAIVGHIVDELRARPETTKYISVQEEWVF
NCU04156.1	LEQRLESFYVFDTEHLHDLSKRAISAHGNDTKAIVKYIVDELNDRNGVAPYVNNDEEWVF
FG07315.1 	VEQNLEQFYIFDLKHLDDLSKRALAKHGEDTRAVVKHIVDELSEK--NPEHVNVKEEWVF
AN0451.1  	FDARLEQFYIFDHHHLHDLSQRAIAAHGNDTRAIVQYIVSELDEKVG-STHLNKDEEWVF
          	 : .**.**:** .**.:** :*:: **::*:*:* :**.**  :   . ::. .*****

MG08624.1 	NNAGGAMGGMYIIHAS1VTEYLIIFG~-----------------------------TAIG
NCU04156.1	NNAGGAMGAMYIIHAS~ITEYLIIFG~KPHPPARVSQACASRFFRPITNSTLHTTGTAIG
FG07315.1 	NNAGGAMGAMYIIHAS~VTEYLIIFG1-----------------------------TAIG
AN0451.1  	NNAGGAMGAMYIIHAS1ITEYLIIFG1-----------------------------TAIG
          	********.******* :********                              ****

MG08624.1 	TEGHTGRHTADDYFNILTGEQWAYVPGEYEPEVYPAGSVHHLRRGTVKQYKMPEGCFALE
NCU04156.1	TEGHTGRHTADDYFHILTGTQTAYVPGEYEPEVYPPGSVHHLVRGTVKQYRMPESCFALE
FG07315.1 	TEGHTGRHTADDYFHILSGTQLAYVPGEYAPEVYPAGSIHHLRRGDVKQYKMPEGCFALE
AN0451.1  	TEGHTGRHTADDYFNILQGTQLAYVPGSYEPEVYPAGSVHHLRRGEVKQYKMDSSCFALE
          	**************:** * * *****.* *****.**:*** ** ****:* ..*****

MG08624.1 	YARGWIPPMLFFGFADGLSSTLDFPTLWQTTVVTGREMLGNLMLGKF~-----
NCU04156.1	YARGWIPPMLFFGYADTLSSTLDFPTLWRTSVITGREMISNLLKGKF~-----
FG07315.1 	YARGWIPPMLFFGFADGLSSTLDFPTLWDTTRITGREMINNLIKGKL~-----
AN0451.1  	YARGWIPPMLFFGYADTFSSTLDFPTLWATSRITGREMISNLLKGKI2IWESV
          	*************:** :********** *: :*****:.**: **:   .:
```
